# Perceived Stress in Relation to Demographics and Clinical Forms among Patients with Infective Endocarditis: A Cross-Sectional Study

**DOI:** 10.3390/ijerph192114073

**Published:** 2022-10-28

**Authors:** Romualdas Malinauskas, Mindaugas Malinauskas, Vilija Malinauskiene, Vytautas Zabiela

**Affiliations:** 1 Department of Physical and Social Education, Lithuanian Sports University, Sporto 6, LT-44221 Kaunas, Lithuania; 2 Department of Cardiology, Hospital of Lithuanian University of Health Sciences Kaunas Clinics, Eiveniu Street 2, LT-50161 Kaunas, Lithuania

**Keywords:** perceived stress, infective endocarditis, demographics, clinical forms

## Abstract

(1) Background: Infective endocarditis (IE) is a disease of the endocardial surface of the heart, caused by infection of the native or prosthetic valve or an indwelling cardiac device. Apart from IE predisposing risk factors that include heart conditions and medical procedures, the novel trajectories from demographic factors to perceived stress conditions have been under investigation in recent years. The aim of the present study was to evaluate the associations between perceived stress and demographic characteristics as well as clinical forms of IE among survivors of IE in Kaunas, Lithuania. (2) Methods: A cross-sectional study among IE cases (*n* = 135) at the Lithuanian University of Health Sciences Kaunas Clinics Cardiology department during the period 2014–2017 was performed. Data about IE clinical features, sociodemographic characteristics and perceived stress level (Perceived Stress Scale (PSS-10)) upon diagnosis were collected. Package “SPSS 25.0” was used in the statistical analysis. Logistic regression analysis was performed including gender, previous occupation, place of residence and clinical forms of IE in the analysis of perceived stress among survivors of IE. The STROBE checklist for cross-sectional studies was used in this study. (3) Results: Perceived stress was experienced by 54.8 percent of the respondents. In the final model, the OR (odds ratio) of perceived stress for females was 2.07 as compared to men; for rural residents, the OR was 2.25 as compared to urban residents. These results were statistically significant. A tendency for increased OR of perceived stress for low-skilled workers as compared to high-skilled ones and classical IE clinical form as compared to non-classical form was observed, but these results were not statistically significant. (4) Conclusions: The present study is an attempt to focus the attention of IE researchers on the effects of psychological state in the disease development. Differences in perceived stress and some demographic characteristics, as well as tendencies of IE clinical forms, were observed among survivors of IE in Kaunas, Lithuania.

## 1. Introduction

Psychological stress usually occurs when environmental demands exceed the individual’s abilities to perform in an adequate way, and such stress may lead to an increased risk of heart diseases [1]. Perceived stress is a form of psychological stress, which occurs due to a variety of chronic stressful events that lead to psychological and physical changes of such consequence that the person cannot restore homeostasis [2]. Perceived work stress (work-related stress) is one of the most common long-term psychological stresses that have frequently been associated with increased risk of cardiovascular diseases [3].

Excessive or prolonged psychological stress compromises several physiological systems, which might increase susceptibility to disease. Robust evidence from human studies suggests a considerable modulation of the hypothalamic–pituitary–adrenal axis in response to stress, with altered biological functions such as compromised immunity (e.g., impaired humoral and cell-mediated immunity) and increased inflammatory reactivity [4]. Stress raises cortisol levels, which can weaken the immune system if they stay high for too long [4]. Stress can also dysregulate humoral and cellular immune responses to pathogens, increasing risk for infectious illnesses [5]. Prolonged everyday stress is associated with decreased levels of lymphocytes [5]. Chronic inflammation secondary to long-term stress has been causally linked with risk for numerous diseases, including infectious illnesses, cardiovascular disease, diabetes, certain cancers, and autoimmune disease [6]. Chronic low-grade inflammation is implicated in the link between stress and cardiovascular diseases via contributions to the early emergence, progression, and thrombotic complications of atherosclerosis [7]. One meta-analysis [8] “estimated an approximate 50% increase in cardiovascular disease risk associated with high levels of work stress, defined as low workplace control coupled with high demands, inadequate compensation, or organizational injustice” [9].

Social differences in health have been under investigation in recent years [10]. Research has confirmed that persons in so-called lower occupational [11] and residential positions [12], e.g., unskilled workers and residents of rural districts, experience more stress as compared to those in higher occupational positions and city residents. Rural residents meet with occupational challenges that induce perceived stress and affect their health status [12]. Furthermore, workers in lower classes, i.e., with lower educational and occupational status, were more likely to report poor self-rated health, limited physical functioning and long sickness absence, but at the same time were less likely to experience increased stress feelings and burnout symptoms, showing a reversed health gradient [13]. In contrast, another study found that unskilled workers and the unemployed were the most vulnerable groups in terms of psychological health. Chronic stress arose in this study as a risk factor for poor mental health outcomes [14].

The majority of the investigations in epidemiology of cardiovascular diseases investigated the impact of stress on non-communicable diseases (coronary heart disease, arterial hypertension, etc.) [15], and little is known about how perceived stress affects the onset and course of infections in cardiology, e.g., infective endocarditis. One study found an association between stress-related disorders and risk of life-threatening infections, including sepsis, endocarditis, central nervous system infections, and fatal infections of any other origin [16], implying that traumatic stress may exacerbate the severity of future infections.

Infective endocarditis (IE) is a life-threatening disease with high mortality rates [17]. It is the disease of the endocardial surface of the heart, caused by infection of the native or prosthetic valve or an indwelling cardiac device [18]. Infective endocarditis does not have the same noticeable risk factors as coronary artery disease. IE can occur in all age groups, from early childhood to the very elderly. Moreover, it is more frightening, since patients may not have any other cardiovascular disease but contract IE due to predisposing factors causing bacteremia such as unhealthy teeth, intravenous drug use, indwelling catheters, surgical treatment, hemodialysis and other medical conditions [19]. Despite its infective origin, the onset and course of the disease might be related to stressogenic factors [20]. The understanding of IE’s epidemiology and predisposing causes has changed during the past decades with a shift toward older patient age, prosthetic valves, degenerative valve disease, implantable devices replacing rheumatic heart disease [21] and the frequency of classical IE clinical form [22]. Recent research reports substantial discrepancies in the presentation of symptoms and in the course of the disease [23].

Currently, IE may present with a variety of symptoms; it can also present with slow course of the disease and nonspecific symptoms, which can lead to difficult early diagnosis [24]. The classic clinical form presents with fever and progressive heart valve insufficiencies. However, research shows that the prevalence of non-classical clinical IE forms is increasing, as the disease manifests atypically as heart failure or septic, embolic, pulmonary, rheumatic, renal, meningoencephalic, ocular, anemic and thrombocytopenic clinical forms [25].

There is a lack of research directed towards the role of perceived stress in the course of IE. In cardiology units, patients are often exposed to high levels of stress and several psychological complications, which play a special role in cardiac prognosis [26]. Patients’ experience of living with different types of heart disease, e.g., coronary heart disease, heart failure, etc., has been well investigated throughout the last 10 years [1,27]. However, little is known about perceived stress in patients with IE, though it is common [28]. The current study fills this gap in the literature, investigating perceived stress in relation to demographics and clinical forms among patients with IE. The aim of the present study is to investigate the associations of perceived stress with gender, residency area, work type and clinical IE forms in patients with IE. It is the first study investigating perceived stress in relation to demographics (age, occupational position, residency) and clinical forms among IE patients. We hypothesize that among patients with IE, females, rural residents and retired unskilled workers as well as patients with the classical IE form experience more perceived stress upon IE diagnosis.

## 2. Materials and Methods

### 2.1. Study Design

The cross-sectional study of IE patients was conducted at the Lithuanian University of Health Sciences Kaunas Clinics Cardiology department during the period 2014–2017. STROBE checklist for cross-sectional studies (https://www.strobe-statement.org, accessed on 30 September 2022) was used to ensure quality of this study (see Table S1).

### 2.2. Study Participants, Procedure and Demographic Measures

Retrospective analysis of IE cases (*n* = 135) was performed. All 135 cases of IE were analyzed after the permission was given from the Bioethics Center of the Lithuanian University of Health Sciences (BEC-MF-54). Data were collected on IE clinical features, course, complications and pathogens. Seventy-eight males (57.8%) and fifty-seven females (42.2%) were investigated. Ninety (66.7%) patients were from urban areas and forty-five (33.3%) patients were from rural areas. Patients were also divided into two groups according to their work type—there were 59 (43.7%) retired high-skilled workers and 76 (56.3%) retired low-skilled workers.

### 2.3. Outcomes

IE was diagnosed according to the modified Duke criteria [29]. Early diagnosis of IE is complicated by the changing symptoms and clinical manifestation of the disease. Some authors distinguish several clinical forms of IE according to the manifestation of the disease and further development of symptoms, because the onset and course of IE can vary widely [24]. Based on the experience of Lithuanian authors [25], we divided the patients into groups (clinical forms) according to the manifestation of the disease. In addition to the typical form (fever with chills following heart murmurs), atypical clinical forms of IE (heart failure form, pulmonary form, septic form, renal form, anemic form, thrombocytopenic form, meningoencephalic form, rheumatic form, pacemaker form) were identified. The quantitative outcome was perceived stress.

### 2.4. Measures

Perceived stress was assessed using the Perceived Stress Scale (PSS-10). The PPS-10 was chosen because the scale is a global stress measure, and the PSS items are general rather than event-specific [30]. The infective endocarditis experience does not occur in isolation, and it is therefore appropriate to implement a global measure of perceived stress. Using a global measure of perceived stress is useful for screening purposes and for comparing stress levels between infective endocarditis patients and other clinical and nonclinical populations. The PSS-10 has become one of the most widely used instruments for measuring perceived stress [31], with both empirical studies and systematic reviews showing it to have strong psychometric properties in numerous clinical and nonclinical samples [32].

The PSS-10 consists of ten questions and assesses the level of overall perceived stress in subjects [30] and measures thoughts and feelings about stressful events, control, coping, and experienced stress, as well as how often individuals feel or think in a stressful manner. PSS-10 items are designed to measure the degree to which respondents find their lives unpredictable, uncontrollable, and overloading. These three issues have been repeatedly found to be central components in the experience of stress. Respondents indicated how often they had felt or thought in a certain way on a five-point Likert scale (0 = never, 1 = almost never, 2 = sometimes, 3 = fairly often, 4 = very often). The questionnaire was translated into Lithuanian and adapted. The internal consistency of the questionnaire for the present sample was assessed by Cronbach’s alpha coefficient (.88). The tool has been tested and validated in Lithuanian-speaking samples as a unidimensional tool and demonstrated adequate internal consistency reliability (α = 0.90) [33]. The perceived stress questionnaire was administered to IE patients after confirmation of their diagnosis.

### 2.5. Biases

Two researchers separately reviewed data of every case so that errors in data entry when extracted from registries were avoided.

### 2.6. Sample Size

The optimal sample size could not be calculated, as the pre-existing evidence was insufficient. The study size was dictated by the fixed available sample at the Lithuanian University of Health Sciences Kaunas Clinics Cardiology department during the period 2014–2017 (all 135 cases of IE were analyzed).

### 2.7. Quantitative Outcomes

The points of PPS-10 were summed, and the respondents were divided into two groups determined by the median score: low level of perceived stress (under 24 points) and high level of perceived stress (24 or more).

### 2.8. Statistical Analysis

Package “SPSS 25.0” (IBM, Armonk, NY, USA) was used in the statistical analysis. The results of the statistical analysis of the survey are presented in tables and graphs. The analysis presents absolute numbers (*n*) and their percentages (%) and mean values of continuous variables with standard deviation (SD). The χ^2^ (chi-square) test was used to compare the categorical variables and to reveal if differences between groups were statistically significant. The mean values were compared using the Student’s *t*-test because the values of skewness and kurtosis of all study variables were in the range of −2 to 2, i.e., the distributions of all variables did not significantly differ from the normal distribution. The observed results were considered statistically significant at a *p*-value 0.05 or lower. The logistic regression analysis was performed to identify variables associated with perceived stress. Perceived stress as dichotomous measure was included as the dependent variable and independent variables were gender, previous work type (unskilled/skilled), residential area (rural/urban) and IE clinical forms (atypical/classical). The adjusted ORs (odds ratios) along with their 95% confidence intervals (CI) were calculated and the effect of various independent variables on perceived stress was evaluated.

## 3. Results

### 3.1. Participants

Definite IE was diagnosed for 97 patients (71.9%), and possible IE was diagnosed for 38 patients (28.1%). The prevalence of high perceived stress was 54.8% (*n* = 74). The prevalence of the classical IE clinical form was 61.48% (*n* = 83) and non-classical 38.52% (*n* = 52).

### 3.2. Descriptive Data

We investigated the level of perceived stress in patients with IE in relation to gender, work type, residence area and clinical form of IE. Means and standard deviations of the selected variables are presented in Table 1 together with Student’s *t*-test and Cohen’s *d* effect size. The differences in means were significant in gender and work type groups.

### 3.3. Data on Outcome Variables

Table 2 presents the number of cases and percentage distribution of study variables between perceived stress groups. The chi-square (χ^2^) statistic was employed to test for differences between categorical variables and statistical significance among IE patients with low and high levels of perceived stress. Our results indicated that of the low-level stress group, 32.8% were female; there was a significantly higher proportion of women in the high-level stress group (50.0%): *p* = 0.044. Among the low-level perceived stress group 24.6% were rural area residents, and in the high-level stress group 40.5% were rural area residents (*p* = 0.05). Approximately a third (32.8%) of patients in the low-level stress group displayed the classical form of IE, while in high-level perceived stress group 43.2% of IE patients experienced classical manifestation of the disease; these differences were not found to be statistically significant.

### 3.4. Main Results

We performed the binary logistic regression analysis including perceived stress as the dependent variable and gender, work type, residence area and clinical form of IE as independent variables. The results of the logistic regression model are presented in Table 3. The adjusted OR of perceived stress for females was 2.07; 95% CI 1.00–4.26 as compared to males. For rural area residency, the adjusted OR was 2.52; 95% CI 1.03–4.91 among patients with IE as compared to urban area residents. Low-skilled work type showed a tendency for high perceived stress levels as compared to high-skilled work type. Finally, the classical clinical form of IE was associated with a 1.75-time increase in perceived stress levels as compared to non-classical forms of IE, though those differences were statistically insignificant.

Table 4 shows data on the distribution of perceived stress among the clinical forms of IE. The results indicate that neither the classical clinical form of IE nor the atypical form differ significantly in the prevalence of perceived stress.

## 4. Discussion

Infective endocarditis is an inflammatory disease of the heart, an infection of the endocardium that usually involves valves. The etiology of the disease is almost certainly infection due to bacteria, fungi, etc. Nevertheless, in the pathogenesis of the disease there are predisposing factors that might cause an infectious agent to enter the body, beginning an infectious process due to impairment in immunological protective mechanisms of the organism.

Links between the central nervous stress system and peripheral immune cells in lymphoid organs have been detailed through 50 years of intensive research. The brain can interfere with the immune system, where chronic psychological stress inhibits many functions of the immune system. In recent years, it has been observed that psychological stress can be disease permissive, as in chronic inflammatory diseases, cancer, cardiovascular diseases, acute and chronic infections, sepsis, asthma and others [34]. Current science requires the discovery of pathophysiologic mechanisms linking the brain to periphery to understand the association between the psychological and the physical. An organism’s response to stress is triggered by its nervous system. The hypothalamus receives afferents from several neural structures involved in the response to stress. The hypothalamus in turn activates the sympathetic nervous system, and (through the anterior pituitary gland) the hypothalamic–pituitary–adrenal axis, leading to increased cortisol levels in humans. Increased cortisol has many somatic effects, including increased insulin resistance, central redistribution of adiposity, increased blood pressure and impaired immune response [35].

There are numerous studies that analyze the occurrence of perceived stress and its connection with various diseases. Studies have established the especially significant role of stress perception on CVD disease severity and later course [36]. In cardiology, many studies have investigated the associations between perceived stress and coronary heart and cerebrovascular disease [36,37,38], and evidence of the associations has been supported by a large prospective longitudinal observational cohort study [39]. However, there is a lack of research identifying the links of perceived stress and infective endocarditis in different groups of patients in relation to demographic factors and clinical forms of IE.

The aim of the present study was to investigate the prevalence of perceived stress in IE patients in relation to gender, occupational position and area of residence, as well as clinical form of IE. It is the first study on perceived stress among IE patients. The study found significant associations between perceived stress and gender and residency in rural areas. Therefore, the first part of our hypothesis was supported, as was the case in similar studies with patients who suffered from cardiovascular diseases [40,41]. Women with IE experienced more stress as compared to men, as did rural residents as compared to those dwelling in the cities. Our results support the conclusions of other researchers, who found that women are more prone to stress reactions and that there are social differences in health, with those in lower social positions and living in rural districts having poorer health outcomes than their counterparts.

Specifically, several studies have highlighted that on average women report higher chronic stress and somatic symptoms than men, even when exposed to the same stressors [40,41]. Perceived stress refers to the degree to which an individual views his or her life as stressful; it differs from the actual feeling and symptoms of stress itself, and women have been shown to be more sensitive to everyday stressors [30]. A study among young university students indicated that overall, females experienced higher levels of stress than their male counterparts. Gender differences were evident both in coping dimensions and individual coping strategies used [42]. Furthermore, women were more vulnerable to repeated stress exposures than men [43].

Results from the current study, showing differences in perceived stress between rural and urban areas, are contradictory to some other findings. Rural residents often meet with occupational challenges that induce stress, because those challenges (e.g., weather conditions) are unpredictable [12]. However, one study among young adults indicated that residents in rural areas experienced less perceived stress than those living in urban areas [44]. In contrast, our study confirmed our hypothesis that rural residents with IE diagnosis experienced more perceived stress, and these results are supported by a study among rural women suffering from coronary heart disease that found that living in a rural area was related to a more expressed stress pattern [45].

Finally, another part of our hypothesis was only partially confirmed, as we did not find consistent associations between perceived stress and occupational groups. Instead, only tendencies suggesting an association were found. This may have been because the participants were assigned to low-skilled and high-skilled workers’ groups retrospectively, as they were retired. Although IE may affect people at any age, during recent decades researchers have observed a shift in prevalence toward older patients [46]. The epidemiology of the disease has evolved in recent decades with a doubling of the average patient age [47]. The elderly, who are the most affected patient population, often have many comorbidities and have poorer health reserves and are therefore more susceptible to severe illness than previous cohorts [48].

Currently, IE may present with a variety of symptoms. It may present as an acute and rapidly progressing infection with severe fever; it can also present more slowly, as sub-acute with low-grade fever and nonspecific symptoms, which may prevent a successful early diagnosis. There is substantial discrepancy in the presentation of symptoms and in the course of the disease, which arouses concern for healthcare professionals. The prevalence of clinical forms of IE in Kaunas University clinics between 2002 and 2005 was also studied, and it was found that only 47.4% of IE patients during this time manifested the disease in a typical form. In other cases, IE began in atypical forms (heart failure, septic, embolic, pulmonary, rheumatic, renal, meningoencephalic, ocular, anemic, thrombocytopenic) [25]. It is still not known why IE changed its clinical course, shifting from the classical form to more common atypical forms. We investigated the differences in perceived stress between classical and atypical forms among patients with IE and found no statistical differences in perceived stress levels.

Over the last decade, numerous studies have confirmed the critical role of perceived stress on cardiovascular disease severity and later course [36]. Evidence suggests that mental diseases and coronary heart disease appear to have a shared etiology, including biological, behavioral, psychological and genetic mechanisms [49]. During the same period, a new term, psycho-cardiological disease, emerged in the scientific literature [50].

Infective endocarditis remains an illness that carries a significant burden to healthcare resources. As such, attempts to uncover new predisposing pathogenetic factors are welcome in epidemiology. Recent investigations and a review into IE pathogenesis took into consideration that psychological factors such as stress, depression, anxiety and some personality traits could negatively influence IE development [51]. Patients diagnosed with IE often feel anxiety and fear surrounding their health and life, as it is a traumatic and life-changing diagnosis. Data show that during hospitalization, IE patients must cope with physical suffering and emotional distress which may leave patients with a changed perception of themselves and their bodies [28]. It is well established that many patients admitted to emergency departments for acute coronary syndrome (ACS) experience acute stress that leads to the development of post-traumatic stress symptoms (PTSS) [52]. Stress and fear of dying were associated with occurrence of PTSS in patients hospitalized with acute coronary syndrome [53]. Psychological methods targeting threat perceptions may reduce PTSS and improve clinical course of the disease [54].

“Given that early responses to trauma may be associated with deleterious outcomes, it may be useful to examine acute posttraumatic responses more broadly as a general marker of risk for trauma-related health problems” [20], p. 108. Only one study exists on PTSD (post-traumatic stress disorder) among patients with IE, and the study concluded that 11% of IE survivors had symptoms of PTSD one year after hospital discharge [55]. The qualitative study researched ten patients with IE. Using semi-structured interviews, it was revealed that for the IE patients, a “sudden unexpected physical change occurred that is difficult to understand and interpret” [28], p. 128. The study also found that “during the hospital admission, time is spent thinking about choices and lost possibilities before admission” [28], p. 129. Patients spoke about “interrupted living, which is being ripped out of their daily living” [28], p. 129. These results suggest that patients in the hospital can feel excluded from life, and heart disease can prevent them from continuing their roles within their family, social circles and professions. The study showed how IE can be perceived as an intermezzo in life. “It is a time of uncertainty and impenetrableness where much is changed, not least the perception of the body and self” [28], p. 126.

In another qualitative study, it was concluded that IE patients felt physically weak and mentally imbalanced to varying degrees. Patients considered the uncertainty of recovery trajectory and future capacity to be stressful [19]. The limited research on the psychological state of IE survivors suggests a new direction in CVD epidemiology—to search for novel psychological factors associated with IE onset, course and aftermath of the disease.

### Limitations and Future Directions

This study attempted to reveal perceived stress differences between demographical groups and IE clinical forms among patients with IE. As a challenging study, it certainly has limitations. These limitations arise from the study design. Cross-sectional study design does not allow for revealing the causal relationships between variables, it only underlines a path and directions for future investigations. Another limitation concerns the questionnaire used. Perceived stress captures the stress in everyday life, but in IE studies some other types of stress might be investigated, for example, acute stress disorder or PTSD following 3, 6 and 12 months after developing the disease. In addition to this, other adverse psychological states might also be investigated, e.g., symptoms of anxiety and depression, as well as the copying strategies that individuals use in dealing with stress. As previously mentioned, this is the first study of its kind and future studies on psychological and psychiatric states of individuals with IE are needed, along with the advisement of psychological help for patients in need. As IE presentation is shifting from classical to non-classical forms and the prevalence of atypical IE clinical forms is increasing, research for novel IE predisposing factors should be continued with the purpose of revealing the determinants of classical and atypical IE clinical forms. Despite the limitations mentioned above, this study is an attempt to focus the attention of IE researchers on the effects of psychological state in the disease development.

## 5. Conclusions

The present study breached a literature gap in perceived stress among IE patients. It confirmed the necessity of future research into the topic. The study results indicated that among IE patients, women experienced more perceived stress than men, and rural residents experienced more perceived stress than urban residents. A tendency for perceived stress was prevalent in retired low-skilled workers, as compared to retired high-skilled workers. From a clinical viewpoint, patients with the classical form of IE had a higher probability of increased perceived stress than those with non-classical or atypical IE forms. This study outlines the necessity of future research into perceived stress, as well as stress prevention measures, among IE patients.

## Figures and Tables

**Table 1:** Average scores of perceived stress among patients with infective endocarditis (*n* = 135) according to gender, work type, residence area and clinical form of IE.

Patients	M ± SD	*t* Value	Cohen’s *d*
Males (*n* = 78)	24.68 ± 5.43	−2.72 **	0.48
Females (*n* = 57)	27.40 ± 6.17		
Retired high-skilled workers (*n* = 59)	24.39 ± 5.44		
Retired low-skilled workers (*n* = 76)	26.95 ± 6.02	2.55 *	0.45
Urban area (*n* = 90)	25.46 ± 6.02	−1.04	0.18
Rural area (*n* = 45)	26.58 ± 5.60		
Non-classical clinical form of IE (*n* = 83)	25.51 ± 5.61		
Classical clinical form of IE (*n* = 52)	26.35 ± 6.33	0.81	0.14

Notes. M—mean; SD—standard deviation; IE—infective endocarditis; * *p* < 0.05; ** *p* < 0.01.

**Table 2:** The distribution of study variables between stress levels groups (*n* = 135).

Variables	Low Levels of Perceived Stress		High Levels of Perceived Stress		χ^2^ (df = 1)	*p*
	n	%	n	%		
**Gender**						
Males (*n* = 78)	41	67.2	37	50.0		
Females (*n* = 57)	20	32.8	37	50.0	4.06	0.044
**Work type**						
Retired high-skilled workers (*n* = 59)	30	49.2	29	39.2		
Retired low-skilled workers (*n* = 76)	31	50.8	45	60.8	1.36	0.244
**Residence area**						
Urban area (*n* = 90)	46	75.4	44	59.5		
Rural area (*n* = 45)	15	24.6	30	40.5	3.84	0.050
**Clinical form of IE**						
Non-classical clinical form of IE (*n* = 83)	41	67.2	42	56.8		
Classical clinical form of IE (*n* = 52)	20	32.8	32	43.2	1.54	0.214

Note. When χ^2^ (df = 1) ≥ 3.84, then *p* ≤ 0.05.

**Table 3:** The associations between gender, work type, residence area, clinical form of IE and perceived stress among patients with infective endocarditis (*n* = 135): results of the logistic regression model.

Variables	Adjusted OR	95% CI	*p*
**Gender**			
Males (*n* = 78)			
Females (*n* = 57)	2.066 *	1.00–4.26	0.049
**Work type**			
Retired high-skilled workers (*n* = 59)			
Retired low-skilled workers (*n* = 76)	1.523	0.75–3.11	0.248
**Residence area**			
Urban area (*n* = 90)			
Rural area (*n* = 45)	2.252 *	1.03–4.91	0.041
**Clinical form of IE**			
Non-classical clinical forms of IE (*n* = 83)			
Classical clinical form of IE (*n* = 52)	1.754	0.84–3.68	0.137

Note. IE—infective endocarditis; OR—odds ratio; * *p* < 0.05.

**Table 4:** The associations between gender, work type, residence area, clinical form of IE and perceived stress among patients with infective endocarditis (*n* = 135).

Clinical Forms of IE	Low Levels of Perceived Stress		High Levels of Perceived Stress		χ^2^ (df = 1)	*p*
	*n*	%	*n*	%		
Classical	20	38.5	32	61.5	2.77	*p* > 0.05
Heart failure	12	44.4	15	55.6	0.33	*p* > 0.05
Pulmonary	8	66.7	4	33.3	1.33	*p* > 0.05
Embolic	6	60.0	4	40.0	0.40	*p* > 0.05
Septic	5	50.0	5	50.0	0.00	*p* > 0.05
Other forms	10	41.7	14	58.3	0.67	*p* > 0.05

Notes. When χ^2^ (df = 1) > 3.84, then *p* < 0.05. Other forms—ocular, anemic, rheumatic, pacemaker, renal, meningoencephalic.

## Data Availability

The datasets collected and analyzed during the current study are available from the corresponding author on reasonable request.

## Supplementary Materials

The following are available online at https://www.mdpi.com/article/10.3390/ijerph192114073/s1, Table S1: STROBE Statement—Checklist of items that should be included in reports of ***cross-sectional studies***.
